# Traits influence detection of exotic plant species in tropical forests

**DOI:** 10.1371/journal.pone.0202254

**Published:** 2018-08-22

**Authors:** Decky I. Junaedi, Michael A. McCarthy, Gurutzeta Guillera-Arroita, Jane A. Catford, Mark A. Burgman

**Affiliations:** 1 Centre of Excellence for Biosecurity Risk Analysis (CEBRA), School of Biosciences, The University of Melbourne, Parkville, Victoria, Australia; 2 School of Biosciences, The University of Melbourne, Parkville, Victoria, Australia; 3 Cibodas Botanic Gardens, Indonesian Institute of Sciences (LIPI), Cipanas, Cianjur, West Java, Indonesia; 4 Biological Sciences, University of Southampton, Southampton, United Kingdom; 5 Fenner School of Environment and Society, The Australian National University, Canberra, ACT, Australia; 6 Centre for Environmental Policy, Faculty of Natural Sciences, Imperial College London, South Kensington Campus, London, United Kingdom; Helmholtz Centre for Environmental Research - UFZ, GERMANY

## Abstract

Detecting exotic plant species is essential for invasive species management. By accounting for factors likely to affect species’ detection rates (e.g. survey conditions, observer experience), detectability models can help choose search methods and allocate search effort. Integrating information on species’ traits can refine detectability models, and might be particularly valuable if these traits can help improve estimates of detectability where data on particular species are rare. Analysing data collected during line transect distance sampling surveys in Indonesia, we used a multi-species hierarchical distance sampling model to evaluate how plant height, leaf size, leaf shape, and survey location influenced plant species detectability in secondary tropical rainforests. Detectability of the exotic plant species increased with plant height and leaf size. Detectability varied among the different survey locations. We failed to detect a clear effect of leaf shape on detectability. This study indicates that information on traits might improve predictions about exotic species detection, which can then be used to optimise the allocation of search effort for efficient species management. The innovation of the study lies in the multi-species distance sampling model, where the distance-detection function depends on leaf traits and height. The method can be applied elsewhere, including for different traits that may be relevant in other contexts. Trait-based multispecies distance sampling can be a practical approach for sampling exotic shrubs, herbs, or grasses species in the understorey of tropical forests.

## Introduction

Invasive exotic plant species are a priority for environmental management because they threaten native biodiversity [1] and alter ecosystem processes [2]. Once exotic species are established and naturalised, their management can be very costly [3], and the effort needed for eradication increases markedly with species abundance [4]. For these reasons, early and strategic invasive species management is important, and effective detection of exotic species is key to this endeavour.

Information about exotic invasive species detectability supports decisions at different management stages, from early detection for potential control or eradication, to evaluation of eradication or containment. Early detection of exotic species significantly reduces the cost of management [5], given that preventative management is often more efficient than curative management [6]. Given limited available resources for exotic species management [7, 8], the ratio of detection cost to the benefit of eradicating / managing these species is crucial when aiming for efficient invasive species monitoring [9].

Detectability refers to “the quality of being detectable” [10] or the extent to which something is observable. At the species level, detectability can be measured by the probability that the *species* is detected at a site under a prescribed search strategy (e.g. half an hour of unstructured search), given its presence. Detectability can also be defined at the level of individuals, e.g. the probability than an *individual* is detected along a line transect based on its (perpendicular) distance from the transect. Accounting for detectability provides various benefits to invasive exotic species management. First, imperfect detection can be accounted for in models that estimate the occurrence and abundance of species [11–13] and communities [14–16], leading to more reliable information to guide decisions about suitable management actions [17]. Second, estimates of detectability can guide optimal survey effort allocation and strategies for species detection given limited resources [9, 18–20].

Vegetation surveys use various methods, including unstructured searches [21], fixed plots, plotless (point-based) methods and nearest neighbour techniques [22]. Distance sampling is another potential approach for surveying plant species. Distance sampling is a method for estimating species abundance that uses data on distance to detected targets collected along line transects, and accounts for imperfect detection by modelling how the probability of detecting an individual decays with its perpendicular distance from the transect [23]. The method has been primarily used to survey animals so far, but can be practical for plant surveys too [24]. Being plotless, it can be simpler to implement than plot-based approaches [25], and yet can be as efficient [26]. Apart from its value for abundance estimation, the detectability information from distance sampling can guide survey effort for rare or exotic plants. Lastly, this method is also suitable for individual level detectability, while several recent studies in tropical ecosystem were mostly conducted for or related to species or community level detectability [27–29].

A number of factors can affect plant detectability. For instance, Chen et al. [11] show how site characteristics (e.g. elevation) and survey methods affect the detectability of vascular plant species in Switzerland. Garrard et al. [12] modeled the detectability of plant species in an Australian temperate grassland and showed that the time it takes to detect the first individual of a target species is explained by site characteristics, species abundance, and surveyor experience. In addition, Garrard et al. [12] showed that traits, such as color and flowering time, affect detectability. Indeed, flowers and fruits can be important cues for plant detection, but reproductive organs are not always present when field surveys are conducted. Further study is needed to assess the potential influence of other traits on plant detectability, as well as to expand the scope of detectability studies to other ecosystem types such as tropical forests.

Plant height, leaf size and leaf shape are plausible candidates for explaining variation in detectability among species. These characteristics may be important for detecting invasive plant species, especially in tropical rainforest with dense and complex vegetation structure. Leaf shape, leaf area and plant height are possibly important when distinguishing exotic from native plant species. For example, assuming surveyor can reliably distinguish exotics from natives, it is logical that taller individuals will be easier to detect than shorter ones, especially in dense stands of vegetation like those found in the lower layers of tropical rainforest. Similarly, leaves with distinct shapes or sizes may be easier to detect.

This study tests whether common leaf visual characteristics and plant height correlate with the detectability of individuals of multiple exotic species when surveyed using the distance sampling method in tropical secondary rainforests. Identifying traits that affect plant detectability in general is useful because borrowing explanatory power across species can: (1) increase the accuracy of abundances estimates for species that are detected rarely; and (2) help estimate the effort required to detect a species, even if data on detectability for that species is sparse. The innovation of this study lies in the multi-species detection model where the distance-detection function depends on leaf traits and height, which could serve such applications elsewhere.

## Materials and methods

### Locations

Line transect surveys were conducted in native Indonesian rainforest ecosystems located next to Cibodas Botanic Gardens (Cibodas), Kuningan Botanic Gardens (Kuningan), Baturraden Botanic Gardens (Baturraden) and Eka Karya Bali Botanic Gardens (Eka Karya) (S1 Fig). These four botanic gardens have different ages: Cibodas was established in 1852 and Eka Karya in 1959, whereas Baturraden and Kuningan are considerably younger having been established in 2004 and 2005, respectively.

This field study was approved and permitted by authorities that managed all the accessed lands in this study. These authorities are: Mount Gede-Pangrango National Park (TNGGP) and Cibodas Botanic Gardens—LIPI (Cibodas); Nature Conservation Agency (BKSDA) of Bali and Eka Karya Bali Botanic Gardens—LIPI (Bali); Mount Ciremai National Park (TNGC) and Kuningan Botanic Gardens (Kuningan); and Baturraden Botanic Gardens (Baturraden). This study did not collect or sample any protected species or its propagules. The participant that appears in this manuscript has given written informed consent (as outlined in the PLOS consent form) to publish his image.

These botanic gardens (Table 1) contain native and exotic plant species in their living collections, and are all located next to native tropical montane rainforest ecosystems. The surrounding native forest contains various exotic species, some of which appear to have escaped from the gardens. These ecosystems are dominated by families’ characteristic of tropical montane rainforests, including Fagaceae, Euphorbiaceae, Meliaceae and Lauraceae [30–32]. The climate at the four sampling locations is similar, except for Eka Karya which is relatively drier [32, 33].

**Table 1:** Information about the four tropical rainforest study sites in Indonesia, each located next to a botanic garden.

Sampling Location	Adjacent Botanic Gardens	Mountain slope	Coordinates	Total length (min. & max. length) (m)	Survey date	Mean rainfall (mm/year)
Mount Gede, West Java	Cibodas Botanic Gardens	Eastern	S 06^0^44.515’ E 107^0^00.290’	1350 (100–450)	April 2015	4000–5000
Mount Tapak, Bali	Eka Karya Bali Botanic Gardens	Eastern	S 08^0^16.658’ E 115^0^08.985’	900 (50–150)	May 2015	3000–4000
Mount Slamet, Central Java	Baturraden Botanic Gardens	Southern	S 07^0^18.096’ E 109^0^13.905’	450 (100–150)	June 2015	4000–5000
Mount Ciremai, West Java	Kuningan Botanic Gardens	Northern	S 06^0^49.519’ E 108^0^24.317’	650 (150–350)	July 2015	4000–5000

Transect lines for distance sampling were established from the edge of the botanic gardens into the adjacent native rainforest. Mean annual rainfall ranges were obtained from Whitten, Soeriaatmadja [33].

### Distance sampling

Line transect distance sampling surveys were conducted at all four sampling locations, from the border of each botanic garden towards the interior of the adjacent native rainforest, following the method of Buckland [23] (lay out in S2 Fig). Total transect line length across all sites was 3350 m. Different transect lengths were used at each location because of variations in rainforest sizes (constraining transect length), land contour (affecting survey feasibility), and detection rate of the surveyed exotics. We decided to stop the sampling on a particular transect line when detection rates of exotic species on that transect line were low. We considered the detection rate low when we did not detect the same species within 100 m of the last detection along that transect. Records were limited to detections within a 20 m wide strip (10 m perpendicular to either side of the transect lines). This corresponds to a total area of 6.7 ha (3350 m x 20 m = 67000 m^2^): 2.7 ha in Cibodas, 1.8 ha in Eka Karya, 0.9 ha in Kuningan, and 1.3 ha in Baturraden. Perpendicular distances were measured using a Bosch GLM50 laser rangefinder.

### Leaf shape, leaf size and plant height

Our candidate species traits were leaf size, leaf shape and plant height, with methodology for measuring traits based on Pérez-Harguindeguy et al. [34]. With a limited number of exotic species recorded during the surveys, we limited the number of species traits to reduce the risk of over-fitting in the detection model. While other traits undoubtedly influence detection, we focused on these traits because we expected them to be particularly relevant to detection of exotics species in the understorey. We conducted multiple measurements for each trait (on each individual) and used average values following the suggestion of Pérez-Harguindeguy, Díaz [34]. One leaf sample set was collected from each of the detected plants. Each set consisted of between 1 and 10 leaves (mean ≈ 3–4), depending on leaf availability during sampling. Plant height was defined as “the shortest distance between the upper boundary of the main photosynthetic tissues (excluding inflorescences) on a plant and the ground level” [34]. Traits values may be subject to phenotypic plasticity [35]. However, we did not take account for this and assumed that the sampled trait values reflected a single value for each species.

Along with the exotic species data samples, we also collected digital photo images and data on physical leaf samples from dominant native species. Native species dominance was determined based on written descriptions of the vegetation communities (S1 File). The native species considered dominant were 4 tree species and several understorey species (3 vines, 7 shrubs, and 8 herbs).

We collected healthy, young but fully expanded leaves with no physical, predator, or disease caused defects [34]. We photographed samples next to a scale in a laboratory using a Panasonic Lumix DMC-TZ10 digital camera for leaf area, leaf size and leaf shape calculation (examples for every species in S4 Fig).

Leaf area and complexity data for exotic and native samples were measured by analysing the leaf image in the IMAGEJ image processing program [36]. The distribution of leaf area among species was quite skewed, so we quantified leaf size as the square-root of leaf area; this helped make the distribution of the data more even so the potential for leverage was reduced. The use of the square-root of leaf area, which approximates the average linear dimension of the leaves, is also a natural way to consider the size of a leaf. We quantified leaf complexity as the ‘leaf dissection index’ of McLellan and Endler [37], which is computed as leaf perimeter divided by the square-root of leaf area. High values of leaf complexity indicate more complex leaf shape or extreme width-length leaf ratio. If leaf complexity of exotics is considerably different from natives, then leaf complexity may improve exotic species detectability.

**Fig 1:**
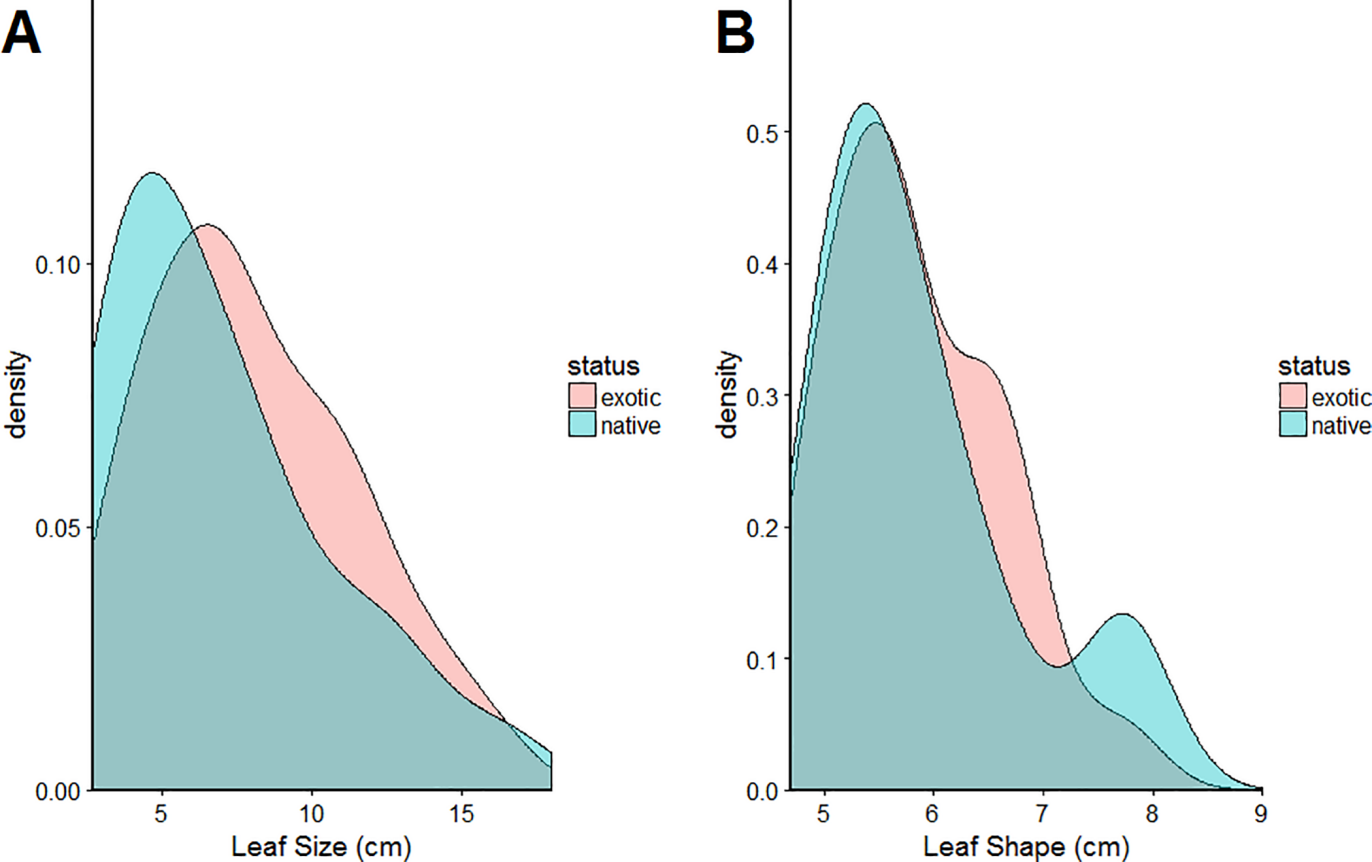
Leaf traits data distribution of exotic and dominant native species collected from four locations: Cibodas, Eka Karya Bali, Baturraden and Kuningan. (A) Distribution of square-root leaf area of native and exotic species. (B) Leaf shape of native and exotic species. Total samples: 1890 samples (1338 samples from 25 exotic species + 552 samples from 22 native species).

Detected exotic leaf sizes were generally similar to natives (Fig 1A); a Welch two-sample t-test of the difference between native and exotic leaf size was not significantly different from 0 (95% confidence interval: [-3.29 cm, 0.93 cm]; p-value = 0.266; sample sizes: exotics 25 species, native 22 species). Similarly, there were no significant differences between exotics and natives leaf shape (95% confidence interval of difference: [-2.41 cm, 0.31 cm]; p-value = 0.125, sample sizes: exotics 25, native 22) (Fig 1).

### Detection model

We used a half-normal detection model to describe how the probability of detection of species *i* at study site *j* declines with *d*, the perpendicular distance from the transect:

$$g_{ij}(d)=\exp\frac{{-d}^{2}}{{{2\sigma }_{ij}}^{2}}$$ Eq 1

This model assumes that detection of the species on the transect line is perfect, and involves a scale parameter *σ*, which controls the width of the detection function. Large values of *σ* imply that the probability of detection remains high even if the plant is far away from the transect; small values imply that detectability decays quickly with distance from the transect.

In our analysis, we truncated the detection function (zeroed beyond 10 metres), to match the field sampling protocol (only detections within 10 meters from the transect were recorded).

We analysed our multi-species multi-site dataset in a single model, with the scale parameter described as a function of study site and species characteristics using a log regression as follows:

$$\log\left(\sigma_{ij}\right)=b_{0j}+b_{S}S_{i}+b_{S2}S_{i}^{2}+b_{A}L_{i}+b_{A2}L_{i}^{2}+b_{H}H_{i}+\varepsilon_{i},$$ Eq 2

where *b*_0*j*_ controls the average detectability across all species at each site (each of the 4 gardens), each of the *b*_*x*_ parameters is a regression coefficient that represents how the corresponding explanatory variable influences detectability, and *ɛ*_*i*_ is a random effect term that allows for un-modelled variation in detectability among species (i.e., variation not accommodated by the explanatory variables). Random effect values were modelled as being drawn from a normal distribution with mean zero and a standard deviation that was estimated from the data. The explanatory variables included were: mean leaf shape (*S*), mean height (*H)* and mean leaf size (square-root leaf area) (*L*) at the species level. Explanatory variables were centered and, to account for possible non-linear effects, we included quadratic terms of *S* (*S*^*2*^) and *L (L*^*2*^*)*. In tropical rainforest ecosystems, native species have particular leaf sizes or shapes ranges due to their response to high rainfall [38]. Therefore, an exotic species with leaves that differ substantially in size or shape from those of natives (either smaller or larger) might be easier to detect, so a quadratic relationship might be expected.

We conducted our analysis in a Bayesian framework using JAGS [39], called from R with package jagsUI [40]. We provide our JAGS code in S2 File. We ran 3 MCMC chains and drew 10,000 samples per chain, omitting the first 5,000 iterations as a burn-in. We assessed convergence of the MCMC chains using the Gelman-Rubin R-hat diagnostic [41]; we assumed convergence when R-hat values were smaller than 1.1. We used uninformative priors for all the parameters. Specifically, for the regression coefficients we used a normal distribution with a mean of 0 and standard deviation equal to 1000 (i.e. precision 1 x 10^−6^). The prior for the standard deviation of the random effect was a uniform distribution on the range 0 to 10. The likelihood function of the distance sampling model [42] is not readily available as a sampling distribution in JAGS, so in our code we implemented it using the “zeroes trick” which allows arbitrary sampling distributions to be used [43]. We limited the analysis to species that were detected at least five times.

## Results

**Fig 2:**
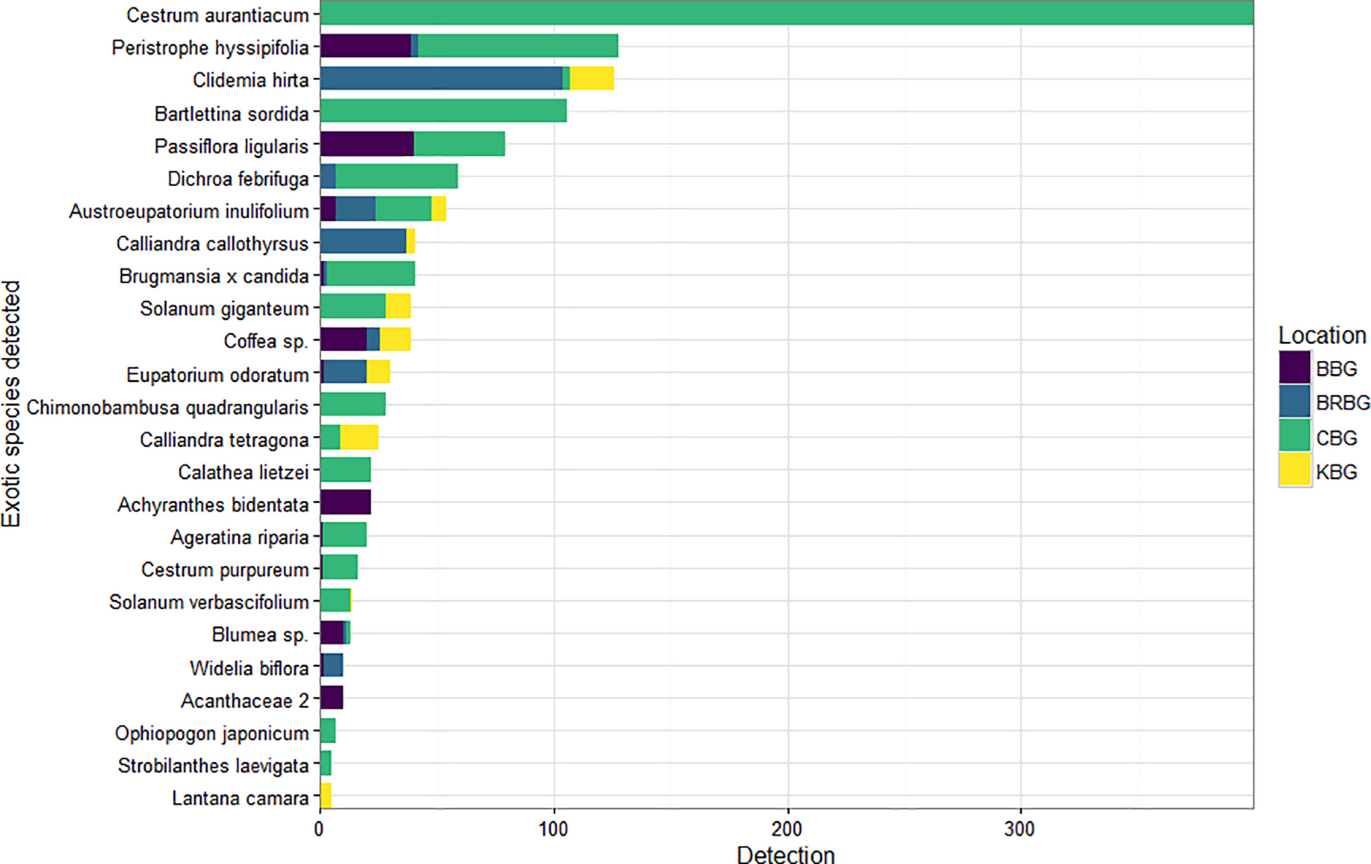
The number of times each exotic species was detected at each of the four locations for the species included in the detectability model. BBG: Bali Botanic Gardens, BRBG: Baturraden Botanic Gardens, CBG: Cibodas Botanic Gardens, KBG: Kuningan Botanic Gardens.

We recorded 1440 individuals in total, detecting 55 exotic species of which 25 had at least five detections. The 30 species omitted for analyses made up only 102 detections. *Cestrum aurantiacum* Lindl. at Cibodas was the species most frequently detected with 399 detections (Fig 2).

We did not detect large exotic tree species in this study, but we detected small exotic trees (*Calliandra callothyrsus* and *Zapoteca tetragona*). In higher elevation tropical forest, trees tend to be smaller than at lower elevations [32]. We are also certain that there are no large exotic trees in the study site because previous studies also noted their absence [44–48].

The probability of detection of individuals increased significantly with height; the regression coefficient *b*_*H*_ was estimated with mean = 0.110 and 95% credible interval [0.028, 0.200] (Table 2), with the detection probability changing by up to 0.4 over the range of the data.

**Table 2:** Summary statistics for the posterior distribution of parameters in the detection model (Eq 1 and Eq 2).

	mean	sd	2.5%	97.5%	R-hat
b_0[1]_ (Location, Bali)	1.197	0.085	1.033	1.364	1.013
b_0[2]_ (Location, Baturraden)	0.989	0.101	0.791	1.185	1.012
b_0[3]_ (Location, Cibodas)	1.34	0.07	1.202	1.481	1.011
b_0[4]_ (Location, Kuningan)	0.804	0.107	0.591	1.011	1.001
b_S_ (leaf shape)	0.012	0.017	-0.023	0.044	1.006
b_S2_ (squared leaf shape)	0	0.001	-0.001	0.001	1.002
b_H_ (plant height)	0.136	0.051	0.048	0.252	1.009
b_A_ (leaf size)	0	0.032	-0.07	0.06	1.012
b_A2_ (squared leaf size)	0.007	0.009	-0.009	0.025	1.016
sd	0.168	0.071	0.041	0.322	1.025

**Fig 3:**
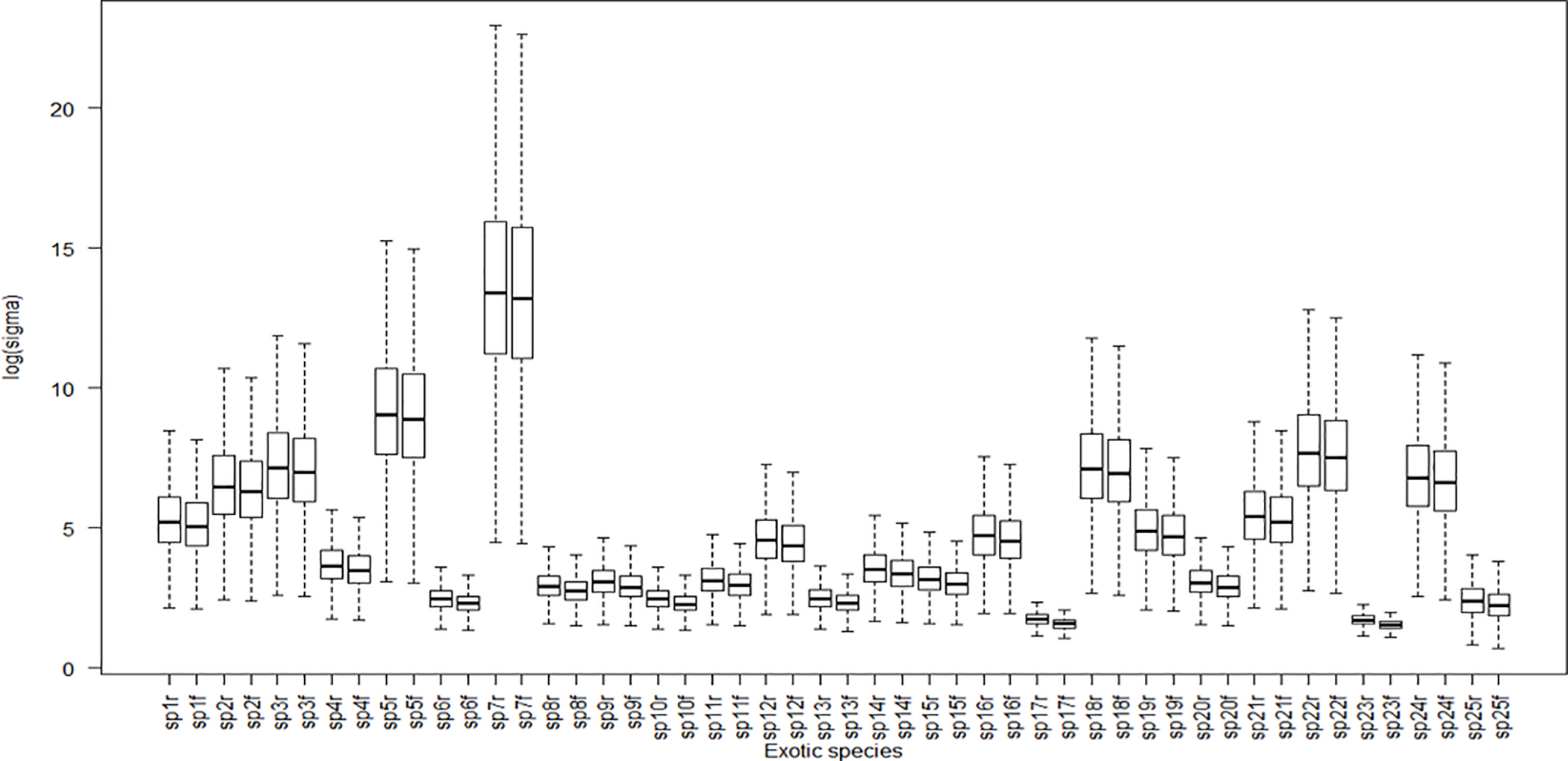
Predicted detection (sigma, in log scale) of 25 detected exotic species. Predicted detection based on fixed model only labelled with ‘f’ (e.g. sp1f). Predicted detection includes random effects in the detectability model labelled with ‘r’ (e.g. sp1r).

Detectability varied substantially among the four locations. Detectability was lowest at Kuningan and highest at Cibodas. The standard deviation of the random effect was not estimated to be particularly large, suggesting that there are modest differences in the detectability of species that remain unexplained by species height, leaf area and leaf shape (Fig 3).

**Fig 4:**
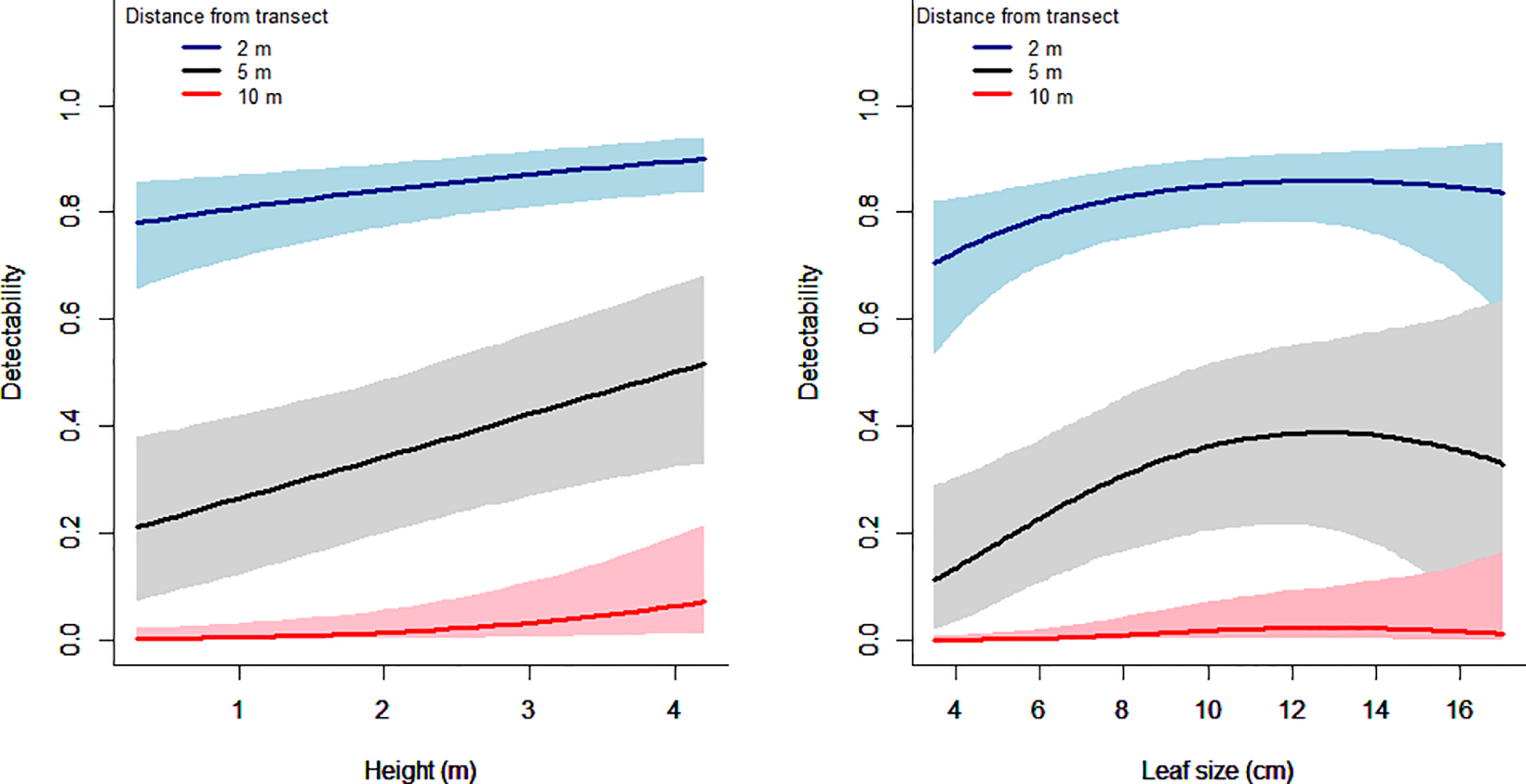
The predicted detectability of exotic species at a particular survey location. (a) Predicted detectability as a function of plant height. (b) Predicted detectability as a function of leaf size. Predicted detectability at different distances (2 m, 5 m, and 10 m) from the transect represented in one curve. The shaded areas show 95% credible intervals.

We failed to detect an effect of leaf shape on detectability; the 95% credible intervals for the coefficient for leaf shape and squared leaf shape include zero (Table 2). However, we did find evidence of a linear effect of leaf size (*b*_*A*_), with detectability generally increasing with leaf size, particularly up to 11–13 cm; the effects were relatively small, with the estimated change in the probability of detection generally being < 0.2 over the range of the data (Fig 4). The credible interval of the quadratic term for leaf size (*b*_*A2*_) included zero, suggesting that the expected relationship between leaf size and detectability (increased detectability of species with extreme leaf sizes; see *Detection model* in the methods) might not exist. Indeed, the estimated effect suggests, if anything, the opposite relationship, with slightly greater detectability at intermediate leaf sizes, although any non-monotonic relationship that might exist appears to be small (Fig 4).

## Discussion

**Fig 5:**
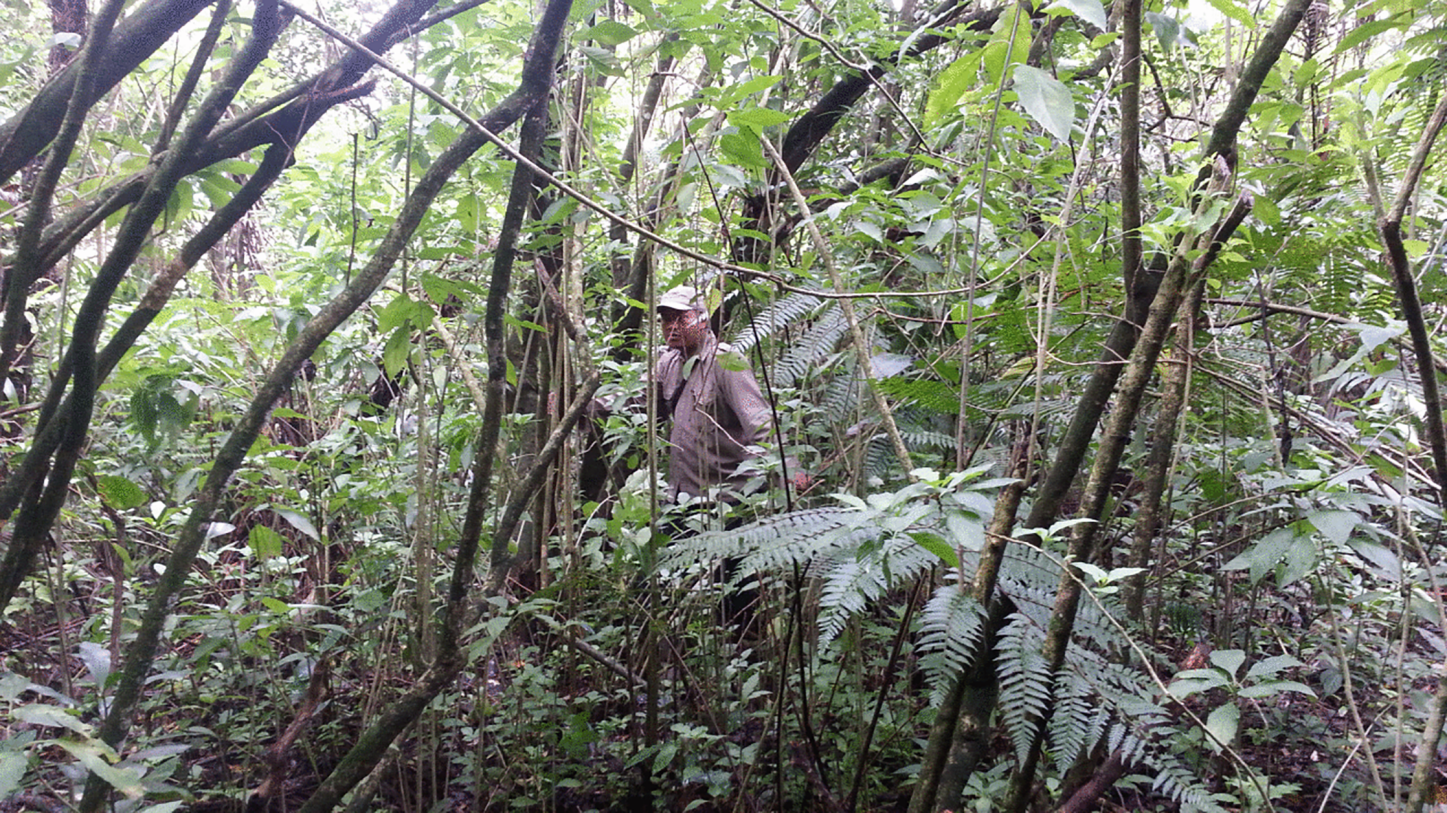
An example of lower layer rainforest (understorey). This native rainforest is located next to Cibodas Botanic Gardens and managed by Gede-Pangrango National Park. Picture was taken in May 2015 by DIJ.

As expected, taller species were detected more easily than shorter species (Fig 4A). A previous study that assessed the effect of plant life form on detectability did not find such an effect [25]. This can be explained because it was a study of detectability at the species level and detection probability of species varies with local abundance [49]. Larger species will tend to be less abundant than smaller species. Therefore, the limited variation in detection probability among life forms in Chen et al. [25] might be due to differences in abundance. In addition, we can explain the important effect of height in our study given that it was conducted in the lower layer of the tropical rainforest, which is dense and filled with numerous different plant species (Fig 5), particularly in secondary tropical rainforest [50]. Tropical rainforest is the most diverse and complex ecosystem type among all tropical land ecosystems [51]. Thus, considering the dense and crowded community in the lower layers of tropical secondary rainforest, it is not surprising that taller exotic species were easier to detect than shorter ones.

The probability of detecting exotic plant species increased with leaf size. This is reasonable given that larger leaves might be more prominent, similar to the effect of plant height. Species with smaller leaves might be more easily obscured by other leaves and simply harder to recognize during surveys. The anticipated effect of species being more easily detected if they had extreme leaf sizes might not exist, perhaps because the range of leaf sizes of exotic species was quite similar to that of native species (Fig 1), so exotic species with extreme leaf sizes might not stand out as being distinctively exotic.

Initially, we expected that exotics with higher leaf shape scores would be easier to detect. However, we did not find evidence for this. There are several potential reasons why leaf shape might not influence detectability. The similar leaf shapes of natives and exotics mean that exotic species might not stand out from natives during surveys. More generally, it may be hard to detect a correlation between leaf shape and detectability because leaf shape varies among and within species [52]. However, similar results were obtained when leaf shape was introduced in the model as an individual level characteristic (unpublished data). Alternatively, other leaf shape measures from morphometric analysis may lead to different results than when using the leaf dissection index. Morphometric analysis might reveal leaf shapes that are more detectable during surveys. Leaf complexity may also be important when combined with leaf colour (i.e. both of them collectively affect detectability). Colour is an important plant attribute for detection purposes [12, 53], but we did not consider it in this study because insufficient data were available.

This study did not consider the spatial distribution of detected exotic species (e.g. randomly distributed vs clumpy distributed; [54]). Detected exotic species in this study might not be distributed randomly, a major assumption in the standard distance sampling method [23]. We also did not include environmental characteristics or surveyor experience, unlike Garrard et al. [12]. We controlled surveyor experience by having the same surveyor for all samplings. In our study, environmental characteristics were similar across sites and the same person conducted all the surveys. We did not consider topographic factors in this study. All four botanic gardens are situated in similar contexts (at the foot of a mountain and within relatively similar elevation ranges and topographic situations) and we assumed that topography did not strongly affect detectability across different study sites. Lastly, height and leaf size themselves might not be the actual reasons for improved detectability, but rather other important traits correlate with them (e.g. showy flowers or fruit color). We focused on leaf traits and height, ignoring flower or fruit as detection cues, because during the observation periods, most of the detected exotics were without flowers or fruits.

### Distance sampling and plant abundance

Distance sampling is often used for animal sampling to estimate abundance [15, 16, 55], but has surprisingly been rarely used for either exotic or tropical rainforest species level vegetation analysis. Distance sampling has similar accuracy to standard survey methods [26], but can be simpler, easier to use and less labour-intensive than standard methods like quadrats or Whittaker’s plots [22]. These standard sampling techniques were primarily developed for use in temperate systems and mostly for multi-species detection [22]. However, standard plot techniques are less suitable and likely to be more laborious and time consuming for exotic plant detection in dense tropical rainforests, which have complex structures and profiles [50]. Our multi-species detection model might be usefully incorporated into distance sampling to help estimate abundance, particularly of rare (and potentially sessile) species.

### Value of traits for exotic plant detectability model

A key value of this study is that it uses information on traits to help predict detectability. The contribution of leaf area and plant height to exotic species detectability are useful for invasive species management. Given trait-based detectability predictions, this study demonstrates that general visual traits (height and leaf size) can be important factors to consider for exotic species survey effort estimation and detection optimization, aside from sampling / survey designs [56–58] and appropriate sampling time [9, 18].

Since most of the detected exotics in this study were shrubs, herbs, and small trees, leaf traits and height significantly contributed to the detectability of these exotic species. We only tested these traits because the detected exotic species were limited to particular life forms, and we aimed to keep the number of traits manageable to avoid over-fitting given the moderate number of species (three traits, 25 species). Other traits may influence detectability, particularly of those species in the higher level of tropical forests. Bark characteristics or branching patterns may be more useful when detecting taller trees. In general, traits that increase the visibility of the species, and traits that help distinguish the exotic species from the natives will be important. Further studies to separate these two factors in the detection model framework would be useful.

Trait-based studies have greatly increased the understanding of invasion processes [59] and for risk assessments [60] in tropical ecosystems, yet have rarely been used for informing exotic species detection in this bioregion. Trait-based detection studies might provide additive solutions for detection optimization. The methodology can be applied to different traits that may be relevant.

## Conclusions

We conclude that vegetative traits such as plant height and leaf size affect exotic plant detection in tropical forests. Even though the effect of leaf size is relatively minor, this trait remains a relevant predictor of exotic species detection in tropical forests. Further study is needed to assess how other potentially relevant vegetative traits, such as branching patterns and wood-bark texture, influence detectability. Our study demonstrates the utility of traits when predicting exotic species detectability, crucial information when determining the required detection effort.

## Supporting information

S1 Fig Map of sampling locations: Cibodas, Kuningan, Baturraden and Eka Karya Bali. (TIF)

S2 Fig Line transect distance sampling lay out. Black circles represent individuals of exotic species and dashed arrows show their perpendicular distance to the transect. Surveys were conducted from the border of the botanic gardens towards the native rainforest interior. Only detections within 10 m of the transect line were recorded. The number of transects, distance between transects and their length varied among locations depending on the field conditions.
(TIF)

S3 Fig History plot of Markov chain Monte Carlo (MCMC) simulation based on the constructed detectability model. The plots demonstrate the converged chains in the model simulation for all variables (nodes) included (*b0*, *bS*, *bS2*, *bH*, *bA*, and *bA2*) (left part of the graph). The credible interval for all involved variables are not containing zero values except for *bA*, *bS* and *bS2*, suggesting weak contribution detected from leaf size (*bA*), shape (*bS*) and its quadratic term (*bS2*) to detectability in this study (right part of the graph).
(TIF)

S4 Fig Examples of used leaf photos for leaf area and leaf shape calculation of 25 detected exotic species. The photo ordered from smallest to the largest leaf shape (complexity) value.
(TIF)

S1 File List of reference publications used for native species composition information data. (PDF)

S2 File R code script for Bayesian analysis of the tested detection model in this study. (PDF)

S3 File Total data set used for the analysis. (CSV)
