# Supplementary figures and images for: Traits influence detection of exotic plant species in tropical forests
Source: PLoS One. 2018 Aug 22;13(8):e0202254. doi: 10.1371/journal.pone.0202254 (PMC6104997; doi:10.1371/journal.pone.0202254)

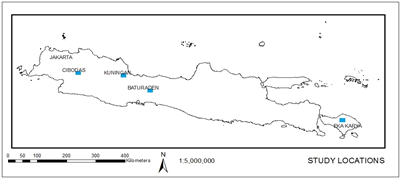

Supplement: S1 Fig — (TIF) [file pone.0202254.s001.tif]

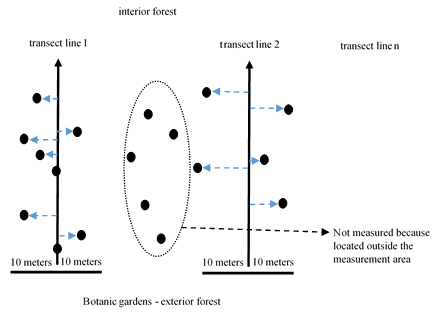

Supplement: S2 Fig — Black circles represent individuals of exotic species and dashed arrows show their perpendicular distance to the transect. Surveys were conducted from the border of the botanic gardens towards the native rainforest interior. Only detections within 10 m of the transect line were recorded. The number of transects, distance between transects and their length varied among locations depending on the field conditions. (TIF) [file pone.0202254.s002.tif]

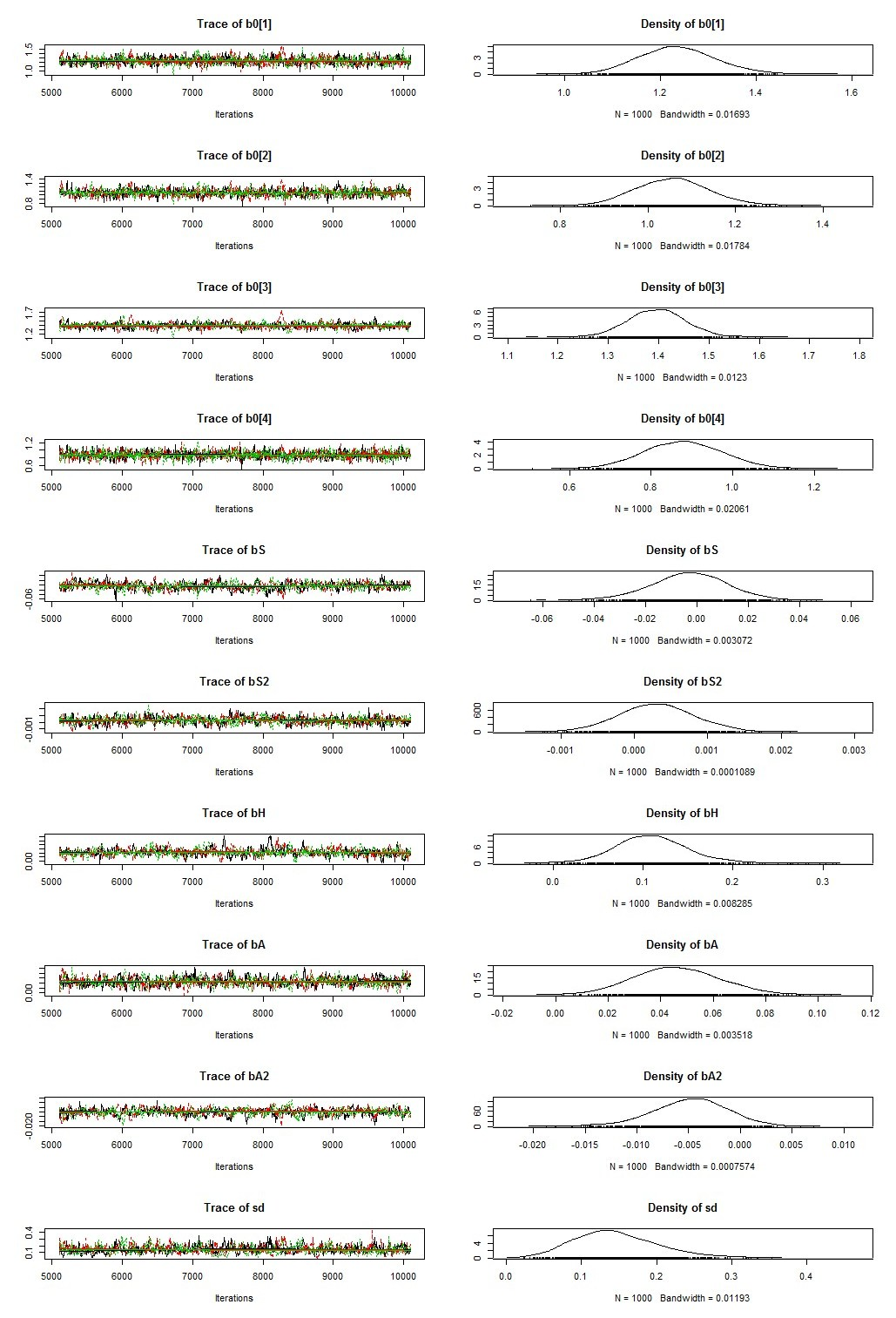

Supplement: S3 Fig — The plots demonstrate the converged chains in the model simulation for all variables (nodes) included (b0, bS, bS2, bH, bA, and bA2) (left part of the graph). The credible interval for all involved variables are not containing zero values except for bA, bS and bS2, suggesting weak contribution detected from leaf size (bA), shape (bS) and its quadratic term (bS2) to detectability in this study (right part of the graph). (TIF) [file pone.0202254.s003.tif]

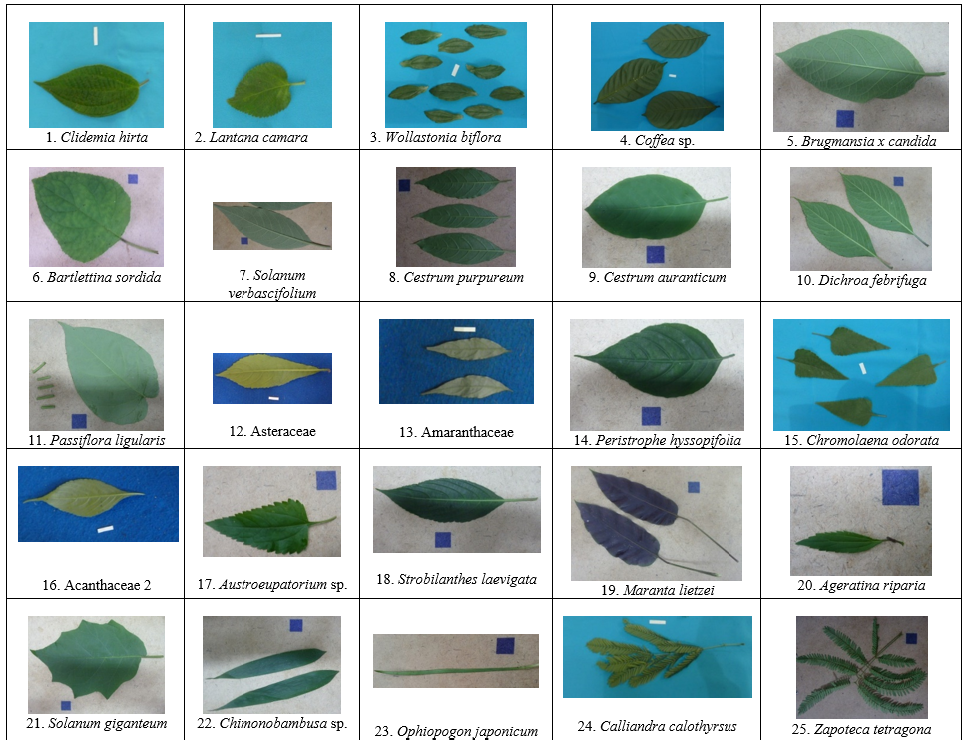

Supplement: S4 Fig — The photo ordered from smallest to the largest leaf shape (complexity) value. (TIF) [file pone.0202254.s004.tif]
