# Supplementary material for: Traits influence detection of exotic plant species in tropical forests
Source: PLoS One. 2018 Aug 22;13(8):e0202254. doi: 10.1371/journal.pone.0202254 (PMC6104997; doi:10.1371/journal.pone.0202254)
Supplement: S1 File — (PDF) [file pone.0202254.s005.pdf]

S1 File. List of reference publications used for native species composition information data

A. Cibodas

1. Van Steenis, C.G.G.J., et al. (1972). "Mountain flora of Java."
2. Werner, W. L. (1986). "A comparison between 2 tropical montane ecosystems in Asia Pidurutalagala (Ceylon Sri-Lanka) and Pangrango-Gede (Java)." Mountain Research and Development **6**(4): 335-344.
3. Yamada, I. (1975). "Forest ecological studies of the montane forest of Mt Pangrango, West Java. 1." Stratification and floristic composition of the montane rain forest near Cibodas. South East Asian Studies **13**: 402-426.

B. Bali

1. Mukaromah, L. (2010). "Autekologi Purnajiwa (Euchresta horsfieldii (Lesch.) Benn.(Fabaceae) di sebagian kawasan hutan Bukit Tapak Cagar Alam Batukahu Bali." Jurnal Biologi **14**(1): -.
2. Rai, I., et al. (2009). "Diversitas flora di Bukit Abah, Kabupaten Klungkung untuk mendukung pengembangan ekowisata." Bumi Lestari **9**(1): 103-111.
3. Siregar, M. and N. K. E. Undaharta (2014). "Vegetasi alami dan perubahannya setelah 22 tahun (1986–2008) di hutan tanaman *Altingia excelsa* Noronha Candikuning-Bali [Natural Vegetation and Its Changes Over 22 Years (1986–2008) in Plantation Forest of Altingia excelsa Noronha Candikuning-Bali]." Berita Biologi **13**(2).
4. Sutomo, S., et al. (2012). "Studi awal komposisi dan dinamika vegetasi pohon hutan Gunung Pohen Cagar Alam Batukahu Bali." Bumi Lestari **12**(2).

C. Baturraden

1. Ardhita, E. O. (2013). "Keanekaragaman Tumbuhan Berguna di Hutan Lindung Gunung Slamet RPH Baturraden, BKPH Gunung Slamet Barat, KPH Banyumas Timur."
2. Van Steenis, C.G.G.J., et al. (1972). "Mountain flora of Java."

D. Kuningan

1. Gunawan, H. "Kondisi Vegetasi Hutan Pinggiran Dan Implikasi Pengelolaannya Di Taman Nasional Gunung Ciremai."
2. Irwan, T. D. (2009). "Komposisi Jenis dan Struktur Tegakan Hutan di Taman Nasional Gunung Ciremai, Jawa Barat."
3. Junaedi, D. I. (2008). "Keragaman komunitas tumbuhan di Taman Nasional Gunung Ciremai." Buletin Kebun Raya **11**(2): 25-32.
4. Rozak, A. H. and H. Gunawan (2015). "Altitudinal gradient affects on trees and stand attributes in Mount Ciremai National Park, West Java, Indonesia." Jurnal Penelitian Kehutanan Wallacea **4**(2): 93-99.
