# Supplementary material for: Traits influence detection of exotic plant species in tropical forests
Source: PLoS One. 2018 Aug 22;13(8):e0202254. doi: 10.1371/journal.pone.0202254 (PMC6104997; doi:10.1371/journal.pone.0202254)
Supplement: S2 File — (PDF) [file pone.0202254.s006.pdf]

S2 File. R code script for Bayesian analysis of the tested detection model in this study.

```
data
    {
    for (i in 1:Ndet)
      {
      zeros[i]<- 0  #here if JAGS
      }
    }

    model
    {
      for (i in 1:Ndet)
      {
        #zeros[i] <- 0  #here if BUGS
        zeros[i] ~ dpois(phi[i]) # likelihood is exp(-phi[i])
        phi[i] <- log(sigma[i]) + log(pi/2)/2 + pow(d[i], 2) / (2 * sigma[i] * sigma[i])
- log(2*phi(10/sigma[i]) - 1) # -log(likelihood) truncation at distance 10 meter (d <
10 meters)
        sigma[i] <- exp(scale[i])
        scale[i] <- b0[Garden[i]] +  re[SpeciesID[i]] # scale parameter of det function
      }

      for (i in 1:Nsp)
      {
        ddd[i] ~ dnorm(0, tau)
        re[i] <- bA*(L[i]-MSA) + bA2*(L[i]-MSA)*(L[i]-MSA) + bS*(S[i]-MSS) + bS2*(S[i]-
MSS)*(S[i]-MSS) + bH*(H[i] - 2) + ddd[i]      #H[i]  substracted  by  2  to  obtain
positive value only for exponential scale
      }
      MSA <- mean(L[])
      MSS <- mean(S[])
      #priors
      pi <- 3.1415926
      b0[1] ~ dnorm(0, 1.0E-6)  # sampling location: Bali
      b0[2] ~ dnorm(0, 1.0E-6)  # sampling location: Baturraden
      b0[3] ~ dnorm(0, 1.0E-6)  # sampling location: Cibodas
      b0[4] ~ dnorm(0, 1.0E-6)  # sampling location: Kuningan
      bS    ~ dnorm(0, 1.0E-6)  # shape (individual level)
      bS2   ~ dnorm(0, 1.0E-6)  # shape squared (individual level)
      bA    ~ dnorm(0, 1.0E-6)  # square root of average leaf area (species level)
      bA2   ~ dnorm(0, 1.0E-6)  # average leaf area  (species level)
      bH    ~ dnorm(0, 1.0E-6)  # species average height
      sd    ~ dunif(0, 10)
      tau <- 1 / (sd *sd)

  }
```
